# Trauma-informed veterinary practice: linking emotional labor, moral distress, and occupational well-being

**DOI:** 10.3389/fvets.2026.1746939

**Published:** 2026-02-02

**Authors:** Begüm Serim-Yıldız, Selin Onaylı, Hüseyin Emre Ilgın

**Affiliations:** 1 Psychological Counseling and Guidance, Faculty of Education, TED Universitesi, Ankara, Türkiye; 2 Faculty of Built Environment, Tampere University, Tampere, Finland

**Keywords:** emotional labor, moral distress, occupational well-being, trauma-informed care, veterinary practice

## Abstract

This study explores the emotional and psychological challenges inherent in veterinary practice, a profession situated at the intersection of empathy, ethics, and trauma. Despite its compassionate reputation, veterinary work frequently exposes practitioners to distressing experiences, including euthanasia, caregiver grief, and moral dilemmas, which can culminate in burnout and compassion fatigue. Drawing on a Trauma-Informed Care (TIC) perspective, this study explores veterinary professionals’ lived experiences and understands occupational distress as a systemic and relational experience rather than an individual shortcoming. By moving beyond individual-level burnout models, veterinary distress is reframed as an outcome of cumulative exposure to suffering, moral conflict, and emotionally charged caregiver interactions embedded within organizational contexts. Using a qualitative phenomenological design, semi-structured interviews were conducted with six veterinary professionals, with data collection concluding upon thematic sufficiency, identifying emotional stressors, coping mechanisms, and unmet psychoeducational needs. Findings reveal (1) persistent emotional exposure and moral conflict as key sources of stress, (2) limited institutional support and a prevailing culture of endurance, and (3) strong reliance on informal peer networks for emotional regulation. These findings underscore the need for trauma-informed education, structured debriefing, and peer support systems within veterinary institutions. For stakeholders, veterinary schools, professional associations, and clinics, adopting trauma-informed policies offers a practical route to mitigating compassion fatigue, enhancing psychological safety, and sustaining compassionate engagement in animal care.

## 1 Introduction

**Figure 1:**
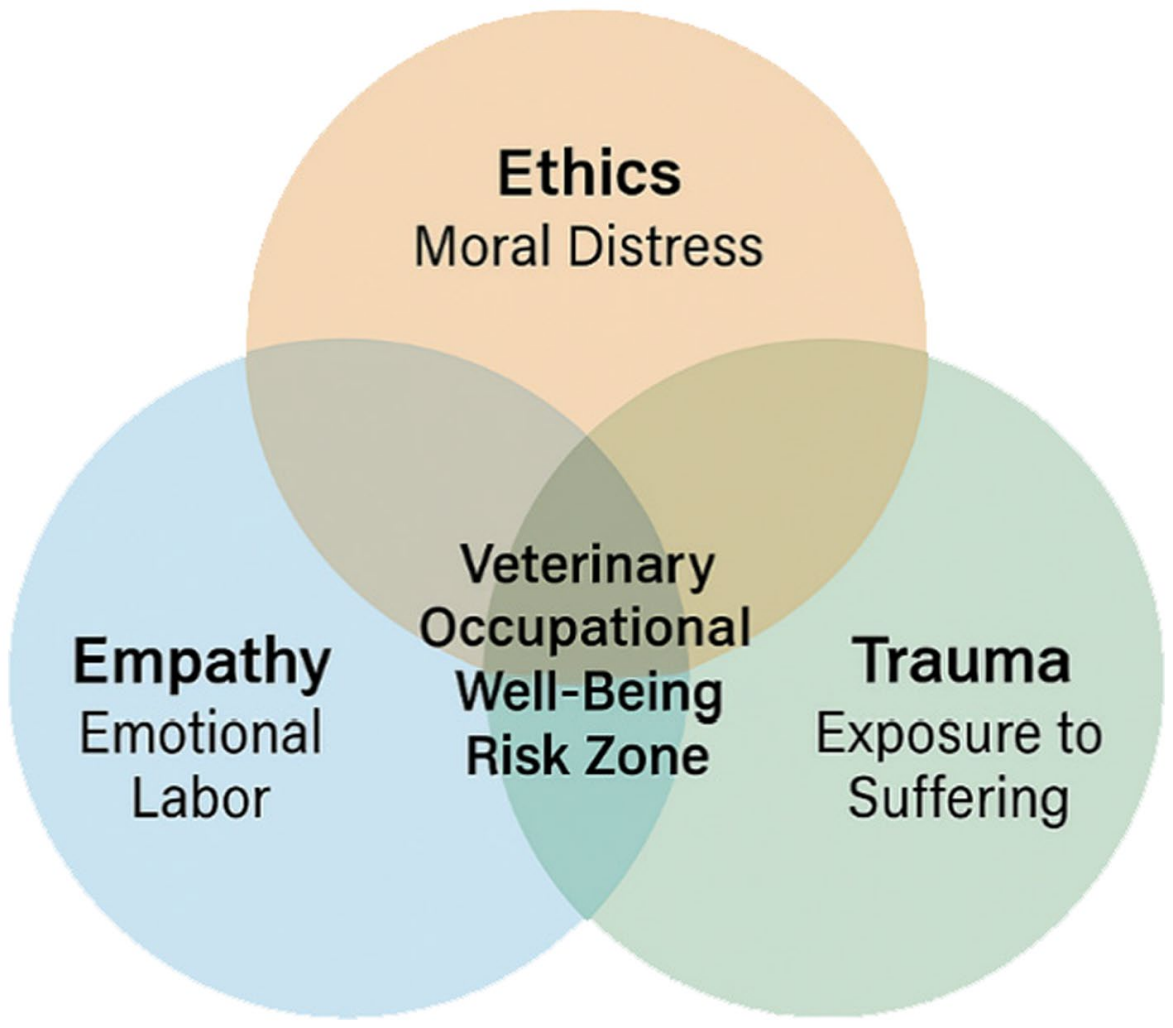
Conceptual intersection of empathy, ethics, and trauma in veterinary practice. The figure illustrates how emotional labor (empathy), moral distress (ethics), and exposure to suffering (trauma) intersect to shape occupational well-being risks in veterinary practice.

The veterinary profession occupies a distinctive position at the intersection of human and animal health, empathy, and crisis (1). Although the field is frequently depicted as emotionally fulfilling, a growing body of research uncovers a far more somber and sometimes ignored reality. Veterinarians frequently encounter considerable psychological stress, ethical dilemmas, and emotional distress arising from their profession (2, 3). These job-related pressures, which include euthanasia, witnessing abuse, having emotionally charged conversations with companion animals’ caregivers, and making ethically constrained decisions, can have a profound impact on mental and emotional well-being (Figure 1). Over time, repeated exposure to such morally and emotionally demanding situations constitutes cumulative occupational strain, increasing veterinary professionals’ risk of burnout, compassion fatigue, depression, and suicide (4–6).

From the standpoint of occupational health psychology, the veterinary profession exemplifies work characterized by high emotional demands, ethical complexity, and limited psychosocial resources. Within the Job Demands–Resources (JD–R) framework, such pressures function as persistent job demands which, when not counterbalanced by adequate organizational resources, lead to emotional exhaustion, disengagement, and moral distress (7, 8). Together, JD–R, COR, and a trauma-informed perspective help to understand veterinary distress as a systemic and relational response to emotionally and ethically demanding work environments, rather than as an individual vulnerability.

Veterinary professionals’ experiences further illustrate the burdens of emotional labor, the regulation of emotions to meet professional expectations (9). They are required to deliver medical care to animals while simultaneously supporting distressed or grieving companion animals’ caregivers, thereby assuming a dual caregiver role (10). This dual responsibility produces what has been described as a “relational load,” in which practitioners absorb both animal suffering and caregiver distress. Repeated exposure to emotionally charged encounters, particularly under financial or organizational constraints, contributes to compassion fatigue and secondary traumatic stress (11, 12), with consequences extending beyond individual well-being to professional sustainability and workforce retention.

**Figure 2:**
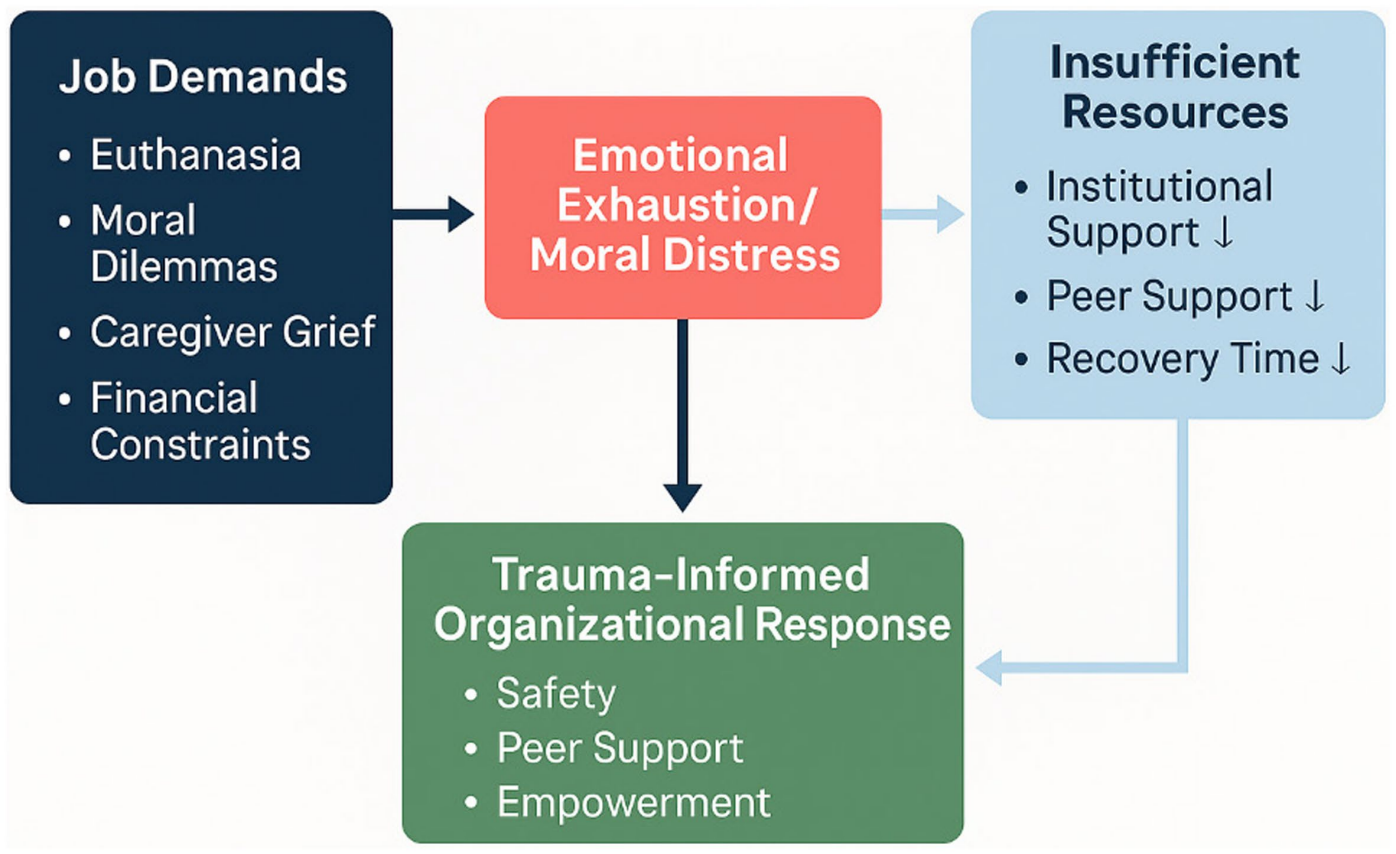
Refined systemic framework of occupational stress and trauma-informed response in veterinary practice, illustrating the interaction among job demands, insufficient resources, and organizational resilience mechanisms fostering safety, peer support, and empowerment.

According to Conservation of Resources (COR) theory (13), such environments foster ongoing resource depletion, as emotional, cognitive, and moral resources are continuously expended without sufficient recovery or institutional replenishment (Figure 2). In this study, COR theory informs both the interview focus (e.g., perceived losses of emotional energy, time, moral agency, and social support) and the analytic interpretation of how veterinary professionals attempt to protect or restore depleted resources.

This study was conducted in Türkiye and focuses on veterinary professionals working in companion-animal (small-animal) clinical settings, a context characterized by particularly intense relational and emotional demands. Companion-animal practice concentrates key dynamics relevant to a trauma-informed perspective, including frequent end-of-life decision-making, direct exposure to caregiver grief, and time-pressured, emotionally charged communication, making it a high-salience setting for examining cumulative occupational trauma. Türkiye represents a critical empirical context due to the rapid growth of companion-animal ownership alongside limited institutionalized mental health supports within veterinary workplaces; despite the presence of professional associations and informal peer networks, standardized systems for psychological debriefing or trauma-informed supervision remain scarce. Financial constraints and limited insurance coverage further intensify moral conflict and emotional labor. While the empirical focus is small-animal practice in Türkiye, the mechanisms examined, emotional labor, moral conflict, resource depletion, and relational trauma exposure, are theoretically transferable to other veterinary domains and caregiving professions, albeit with contextual variation in triggers and organizational conditions.

The absence of formal mechanisms for emotional processing, peer support, or ethical consultation perpetuates a culture of endurance rather than reflection. As a result, many veterinary professionals rely on improvised individual coping strategies instead of organization-supported interventions (14). This pattern aligns with research showing that organizational climate, particularly psychological safety and perceived support, plays a decisive role in moderating the health impacts of emotional labor (15, 16).

Existing research has documented elevated rates of burnout, compassion fatigue, moral distress, depression, and suicide among veterinary professionals, yet these outcomes have largely been interpreted through individual-level psychological models (4, 6, 14). Relatively little attention has been paid to how trauma accumulates through relational and organizational processes, such as repeated euthanasia, exposure to caregiver grief, and ethically constrained decision-making. This theoretical gap motivates the present study.

TIC, originally developed in human healthcare (17), emphasizes safety, trustworthiness, peer support, collaboration, empowerment, and cultural sensitivity. In this study, TIC is analytically integrated with JD–R and COR frameworks to form a unified conceptual model, wherein trauma-informed principles operate as organizational resources that can buffer high job demands and interrupt cycles of resource loss.

Thus, rather than positioning well-being as an individual responsibility, the present study conceptualizes trauma-informed practice as an organizational and relational strategy. This integrated framework guides interview design, analysis, and interpretation, and provides a basis for examining how veterinary professionals perceive, manage, and potentially mitigate occupational trauma within structurally constrained settings.

### 1.1 The hidden cost of compassion in veterinary medicine

Veterinary practice is characterized by a distinctive dual caregiver role that differentiates it from many other healthcare professions. Veterinary professionals are responsible not only for the medical treatment of animals but also for managing the emotional needs and grief responses of companion animals’ caregivers (10). This relational configuration creates a two-tiered emotional system in which practitioners must regulate their own affect while simultaneously responding to caregiver distress. Previous research describes veterinary professionals as “mediators of suffering,” tasked with alleviating animal pain while absorbing intense human emotions (18, 19).

Euthanasia represents a particularly salient manifestation of this dual burden. Although often medically justified, euthanasia frequently becomes a shared traumatic encounter in which veterinary professionals witness acute caregiver grief, guilt, or anger. Such experiences are repeatedly described as among the most emotionally demanding aspects of veterinary work (12, 20). Sustained exposure to emotionally charged interactions, particularly those involving blame, despair, or moral conflict, has been shown to contribute to secondary traumatic stress and compassion fatigue over time (21). Veterinary professionals are positioned at the intersection of animal suffering and caregiver trauma, often with limited opportunity for emotional processing or recovery.

Professional norms within veterinary settings further intensify this burden. Veterinary professionals are expected to demonstrate empathy while maintaining emotional composure, a requirement that necessitates continuous emotional regulation and contributes to cumulative exhaustion (14, 22). When such emotional labor is combined with structural constraints, such as staffing shortages, ethical dilemmas, and financial limitations, risks of burnout and moral distress are amplified. In Türkiye, these challenges are exacerbated by socioeconomic instability and limited access to affordable veterinary care, frequently placing practitioners in ethically constrained situations where optimal treatment is financially unattainable.

An additional layer of complexity arises from companion animals’ caregivers’ trauma responses, which may include denial, shame, anger, or prolonged grief. Veterinary encounters are often experienced by caregivers as highly distressing events, particularly when illness or death is perceived as the loss of a family member (10). Consequently, veterinary professionals are repeatedly exposed to intense expressions of human grief and, in practice, assume informal counseling roles during moments of acute emotional crisis. This sustained relational exposure constitutes a central yet frequently under-acknowledged source of occupational strain in veterinary medicine.

### 1.2 Trauma-informed care (TIC): a framework for relational resilience

Building on Figley’s etiological model of compassion fatigue (11), prolonged empathic engagement with suffering and repeated value–treatment conflicts, such as those arising when financial constraints limit optimal care, can culminate in compassion fatigue and moral distress. TIC originally developed in human healthcare, emphasizes safety, trustworthiness, peer support, collaboration, empowerment, and cultural sensitivity as guiding principles for understanding and responding to trauma exposure (17).

Although initially formulated for human service settings, TIC is increasingly relevant to veterinary practice, where both care providers and care recipients may be affected by repeated exposure to suffering, loss, and ethical conflict. In this study, TIC is applied as an analytic lens to interpret how veterinary professionals perceive, manage, and are affected by emotionally and morally demanding interactions with animals and companion animals’ caregivers, rather than as a standalone theory requiring extensive elaboration.

Despite its conceptual relevance, trauma-informed approaches remain unevenly integrated into veterinary contexts. Existing well-being initiatives tend to emphasize individual self-care, with more limited attention to the organizational and relational conditions through which trauma is produced and sustained. This study therefore uses TIC to foreground the organizational and relational dimensions of occupational strain, positioning trauma-informed principles as potential resources for enhancing relational resilience within veterinary practice.

### 1.3 Gaps in veterinary training and organizational support

Despite extensive evidence highlighting the psychological demands of veterinary work, formal veterinary education continues to prioritize biological sciences and technical competencies over emotional literacy and trauma sensitivity (6, 23). Training related to recognizing trauma responses in companion animals’ caregivers or managing veterinary professionals’ own emotional exposure remains limited, contributing to increased vulnerability to moral distress and compassion fatigue.

At the organizational level, workplace cultures may implicitly valorize endurance and self-denial, discouraging open discussion of emotional strain. Prior research has documented a prevailing culture of endurance and silence around mental health in veterinary and other caregiving professions, often characterized by implicit expectations to “keep going” despite emotional strain (4, 6, 14). The present findings align with this literature, as participants described limited opportunities for emotional disclosure and a normalization of overextension within everyday practice, suggesting that distress is often managed through endurance rather than supported reflection. In the absence of structured supports, such as reflective supervision, peer consultation, or post-event debriefing, emotional burdens are frequently internalized, increasing the risk of burnout and professional disengagement.

Companion animals’ caregivers’ emotional needs are similarly under-recognized within many veterinary organizations. Formal frameworks for grief communication, trauma-informed caregiver management, or referral pathways for distressed caregivers are rarely institutionalized. This organizational omission not only prolongs caregiver distress but also intensifies veterinary professionals’ experiences of isolation and powerlessness. Addressing trauma in veterinary practice therefore requires a dual focus on supporting veterinary professionals and acknowledging caregiver trauma as an integral component of clinical work. The present study directly examines these reciprocal dynamics, situating them within a trauma-informed framework that conceptualizes professional and caregiver well-being as relationally interconnected.

### 1.4 The case for a needs-based TIC model

This study examines a significant yet underexplored dimension of veterinary practice: the reciprocal dynamics of emotional strain and trauma within veterinary professionals – companion animals’ caregiver interactions. While existing research has documented compassion fatigue and moral distress among veterinary professionals, comparatively less attention has been paid to how companion animals’ caregiver trauma intersects with professional well-being in everyday clinical encounters. Using a qualitative approach, this study explores veterinary professionals’ emotional and psychological challenges alongside the unmet relational needs of companion animals’ caregivers as perceived by practitioners. By situating these experiences within a trauma-informed analytic perspective, the study identifies relational and organizational gaps that may hinder compassionate and sustainable veterinary practice.

The research is guided by the following questions:

1. What trauma-related emotional and psychological challenges do veterinary professionals experience in their professional practice?
2. What coping strategies and emotional support mechanisms are currently lacking or underutilized?
3. How can trauma-informed principles be adapted to meet the needs of both veterinary professionals and companion animals’ caregivers?

## 2 Methods

### 2.1 Research context

Türkiye constitutes a pertinent setting due to the rapid growth in companion-animal ownership alongside limited institutional mechanisms for psychological debriefing, ethical consultation, or formal peer-support structures in veterinary workplaces. Additionally, constrained insurance coverage and caregiver financial limitations often lead to ethically constrained decision-making, intensifying moral conflict and emotional labour. Studying this context therefore enables an analysis of trauma-informed needs in environments where emotional demand is high but systemic supports are still emerging. To our knowledge, while informal peer support and professional networks exist, there are no widely standardized, clinic-embedded, or routinely implemented debriefing/supervision pathways across veterinary workplaces; therefore, support is typically *ad hoc* and uneven rather than systematized. The research team approached the study with awareness of their disciplinary backgrounds in psychology and counseling, which informed sensitivity to emotional and relational dynamics in veterinary work. Reflexive memos were maintained throughout data collection and analysis to document assumptions, emotional responses, and analytic decisions. These memos were used during team discussions to minimize interpretive bias and to enhance analytic transparency.

### 2.2 Research design

To explore the psychoeducational needs of veterinary professionals regarding TIC, a qualitative needs analysis was conducted by using semi-structured individual interviews. This approach enabled an in-depth understanding of veterinarians’ emotional challenges, trauma-related experiences in their practices, and their perspectives on potential educational support. The study explored how veterinary professionals’ professional and personal experiences shape their emotional resilience, caregiving roles, and perceived need for trauma-informed practices. A qualitative approach was appropriate as it allowed an in-depth exploration of participants’ perspectives, emotions, reflections, and needs. Moreover, it enabled an understanding of interactions with distressed companion animals’ caregivers, ethically challenging decisions, and the emotional impact of animal suffering. The study aimed to capture veterinary professionals’ lived experiences in depth and detail.

The study was not designed as Interpretative Phenomenological Analysis (IPA) in a strict methodological sense. Rather, it employed an inductive thematic content analysis with phenomenological sensitivity, prioritizing participants’ meaning-making and lived experience while generating themes through inductive coding and constant comparison. Accordingly, the terms “interpretive phenomenological stance” and “phenomenologically informed design” refer to the study’s epistemological orientation (attention to lived experience and meaning), whereas “inductive content analysis” describes the analytic procedure used to develop codes and themes. This unified description is methodologically consistent with the analysis steps reported in Section 2.5.

Therefore, a relatively small, information-rich sample was deemed appropriate to ensure analytic rigor and depth (24). Interviews were conducted with the aim of reaching thematic sufficiency (also described as thematic saturation) within a focused scope. Thematic sufficiency was operationalized through codebook stability: after Interview 5, no substantively new codes related to the central analytic domains (trauma-linked demands, coping/resource protection, and support needs) were identified, and Interview 6 served as a confirmatory case. This pattern supported the adequacy of the final sample for the study aims.

A qualitative, phenomenologically informed design was chosen to capture veterinary professionals’ meaning-making around morally and emotionally demanding encounters and to surface practice-proximal needs for trauma-informed supports. This approach is methodologically congruent with our aim to understand how participants experience, interpret, and regulate trauma-linked events in everyday clinical routines, rather than to estimate their frequency.

### 2.3 Participants

In the present study, there were six veterinary professionals (4 men, 2 women), ranging in age from 25 to 52 years (*M* = 35.2). Participants were recruited through purposive and snowball sampling strategies to ensure diversity in both professional background and work setting. Occupational roles included veterinary technicians, clinicians, laboratory specialists, and veterinary officers, with professional experience ranging from 4 to 28 years. This variation ensured the inclusion of both early-career and highly experienced perspectives. Furthermore, participants reported personal connections to animals through companion animals’ caregivership, including cats, dogs, and horses. One of the participants told of the recent loss of a horse that highlights the intersection between professional and personal experiences of animal care, which refers to the dual caregiving responsibilities.

Inclusion criteria were: being a licensed veterinary professional currently practicing in a companion-animal clinical setting in Türkiye; routine face-to-face contact with companion animals’ caregivers; and ≥1 year of professional experience. Exclusion criteria were: primary practice in large-animal/agricultural, zoo/wildlife, or laboratory-only roles with no routine caregiver interaction. Participants were recruited from private clinics and diagnostic/laboratory services that support companion-animal care.

Sampling strategy and justification. Purposive sampling was used to ensure heterogeneity of roles (technician/clinician/laboratory/veterinary officer) and career stage (early-career to senior). Snowball sampling then extended access to additional clinics and professionals. A small, information-rich sample was targeted to achieve thematic sufficiency within a focused scope; after six interviews, no substantively new codes pertaining to trauma-linked work demands, coping, or support needs emerged, indicating adequacy for the study aims.

Participants were approached via professional networks and direct contact with clinics (email/telephone), and those who agreed were invited to share the study information with eligible colleagues for snowball recruitment. No incentives were provided. To reduce clustering of experiences, recruitment sought representation across different work settings; participants were drawn from multiple service contexts (private clinics and diagnostic/laboratory services), and no more than two participants were recruited from the same workplace. Gender was not used as a primary sampling criterion; however, the final sample composition (male-dominated) is acknowledged as a potential influence on the reported patterns of emotional labor and is addressed as a limitation in Section 4.6.

Confidentiality was safeguarded by assigning pseudonyms (e.g., P1–P6) and removing identifying information from the dataset. All data were stored securely in password-protected files (Table 1).

**Table 1:** Demographics of participants.

Participant	Age	Gender	Occupation/Profession	Experience	Companion animals’ caregivership
P1	25	Male	Veterinary Technician	4 years	2 cats
P2	52	Male	Veterinarian/Dog Trainer	28 years	11 dogs, 10 cats
P3	39	Male	Veterinarian/Clinician	18 years	1 horse (recently lost)
P4	34	Female	Veterinarian/Laboratory Specialist	12 years	1 cat
P5	34	Male	Veterinarian/Veterinary Officer	13 years	1 cat
P6	27	Female	Veterinarian/Clinician	4 years	1 cat

Participants are identified using pseudonyms. Companion animals’ caregivership refers to animals currently or recently cared for by participants.

### 2.4 Data collection

The study received ethical approval from the university’s ethics committee before data collection. Participants received information regarding the purpose of the study, their right to withdraw, as well as how all data will be kept confidential. Before the interviews, each participant gave their informed consent. The ethics approval number and date are provided in the Ethical Approval statement (Approval No: TED University, Human Research Ethics Committee/2024–30; Date: 30.04.2024).

Interviews followed a semi-structured guide piloted with two non-participant veterinary professionals for clarity and face validity. Core domains included: (a) emotionally demanding clinical events (euthanasia, treatment limitation, caregiver confrontation), (b) perceived trauma exposure and moral conflict, (c) resource loss/protection experiences (e.g., depletion of emotional energy, time pressure, moral agency), (d) boundary-setting and coping strategies, (e) availability and gaps in organizational supports (debriefing, supervision, peer networks), and (f) views on trauma-informed training content and delivery. The interview schedule used the same core questions for all participants to ensure comparability; follow-up prompts were flexibly tailored to participants’ roles (e.g., technician vs. clinician) to elicit role-relevant examples while maintaining coverage of the same domains. Interviews were conducted in the participants’ preferred language, audio-recorded with permission, and transcribed verbatim. When quotations are presented in English, they were translated and checked against the original to preserve meaning.

The interviewer maintained a reflexive memo after each interview (context, initial analytic impressions, potential biases) and documented non-verbal cues and interactional dynamics where relevant. Member checking was undertaken by sending each participant a short summary of preliminary interpretations for confirmation or clarification. Given the sensitive nature of discussing distressing experiences, participants were informed that they could pause or discontinue the interview at any time; if any distress arose, they were offered information about available psychological support or referral options through institutional channels.

Interviews were conducted online (e.g., via Zoom) and lasted between 20 and 56 min. Interview protocol included questions on demographic information, self-care strategies, veterinary professionals’ responses to trauma among companion animals ‘caregivers, communication approaches with distressed companion animals’ caregivers, and their views on potential training content related to trauma-informed practice.

### 2.5 Data analysis

The interview data were analyzed using content analysis, following an inductive approach. Initial codes were independently generated by three experts and then compared to ensure consistency and analytic depth. Themes were identified through iterative coding, constant comparison, and integration with relevant literature. To enhance rigor, two additional researchers reviewed the coding framework and verified thematic coherence across transcripts.

We conducted an inductive, phenomenologically oriented content analysis. Transcripts were read holistically, then open-coded line-by-line to capture actions, meanings, and emotions linked to trauma-related demands. Codes were iteratively clustered into categories and candidate themes through constant comparison within and across cases. The codebook evolved reflexively: two researchers drafted initial code families, a third researcher challenged category boundaries (negative-case probing), and the team resolved discrepancies through discussion, privileging conceptual clarity over mechanical agreement metrics. Analytic materials (memos, code iterations, decision logs) formed an audit trail. Data management and coding were conducted securely using spreadsheet-based matrices. Coding consistency was validated through iterative reconciliation: the three primary coders compared code applications after initial independent coding, discussed discrepancies, refined code definitions, and re-applied the stabilized codebook to earlier transcripts to confirm interpretive alignment.

#### 2.5.1 Trustworthiness

Rigor and trustworthiness were established by adhering to the evaluative criteria outlined by Lincoln and Guba (25): credibility, transferability, dependability, and confirmability. Credibility was established by asking participants for confirmation of results, data, and explanations. Transferability was achieved by using purposive sampling, and veterinary professionals from diverse settings were included. For dependability, three independent coders were assigned to code and represent similar codes. The three coders had professional backgrounds. For the conformability, from the data collection process to analysis, all stages were meticulously recorded in an unbiased manner. Moreover, the interview protocol was reviewed and approved by two external professionals, ensuring expert input. To guarantee theoretical coherence and methodological rigor, the emerging themes were then contrasted with previously published works.

Thick description of context and cases supports transferability. Reflexive memos and an audit trail enhanced confirmability. We actively searched for negative/deviant cases that challenged emerging patterns and explicitly reported tensions (e.g., times when informal peer support was absent or ineffective). Finally, a brief peer debrief outside the core team was used to question premature closure and to test the robustness of thematic boundaries. Dependability was further supported by maintaining a decision log (audit trail) documenting codebook revisions and analytic decisions, and by re-checking thematic boundaries against the full dataset after codebook stabilization.

## 3 Results

This section presents findings derived from in-depth interviews with veterinary professionals, focusing on their lived experiences and emotional challenges within daily practice. The results are organized into two overarching themes: (1) Veterinary professionals, addressing psychological, emotional, and structural factors influencing professional well-being, and (2) Companion Animals’ Caregivers, reflecting caregivers’ emotional responses and support needs as perceived by veterinary professionals. Together, these findings illustrate the reciprocal emotional dynamics embedded in veterinary care and highlight areas relevant to trauma-informed practice.

### 3.1 Veterinary professionals

Participants’ accounts revealed a convergence of emotional, relational, and structural stressors characterizing veterinary work. Although no systematic differences emerged based on years of experience or practice setting, financial strain, emotionally demanding interactions with caregivers, and limited institutional support were consistently emphasized. Across interviews, veterinary professionals described ongoing emotional labor and moral strain arising from routine practice, alongside individual efforts to remain professionally functional despite insufficient preparation for the psychological dimensions of their role. The subthemes that come after Sources of Stress, Coping Strategies, and Training Needs show how veterinary professionals’ experiences and ways of being professionally resilient are complex and varied.

#### 3.1.1 Sources of stress

Veterinary professionals’ stress was shaped primarily by sustained emotional and relational demands rather than by clinical complexity alone. Participants consistently described interactions with companion animals’ caregivers as the most emotionally taxing aspect of their work, noting that caregivers’ expectations, anxiety, and emotional reactions frequently transformed routine clinical encounters into emotionally charged situations. These experiences reflected high emotional demands embedded in daily practice, with limited opportunities for psychological recovery. A central source of stress involved expectations of constant emotional availability and blurred professional boundaries. Participants reported frequent non-urgent calls and messages from caregivers, including contact outside working hours, which eroded personal boundaries and contributed to a persistent sense of being “on call.” As one participant noted, “messages keep coming after hours.” Such patterns intensified emotional demands while restricting rest and recovery.

Financial constraints constituted another prominent and structurally embedded stressor. Veterinary professionals described ethically challenging situations in which optimal treatment options were financially inaccessible for caregivers, forcing compromises that conflicted with professional values. One participant summarized this dilemma by stating, “you know the best treatment, but you have to choose cheaper options.” These circumstances generated moral distress and feelings of powerlessness, as practitioners expended emotional energy without the means to resolve these conflicts.

Caregivers’ non-adherence to medical recommendations further amplified emotional strain. Participants expressed frustration when treatments were delayed, medications were not administered, or follow-up visits were missed, often resulting in preventable deterioration in animals’ conditions. One veterinary professional remarked that caregivers sometimes “forgot the medication,” leaving practitioners to manage both worsening clinical outcomes and emotional exhaustion. Such interactions compounded cumulative job demands by requiring additional emotional regulation during already strained encounters.

Strong emotional identification with animals emerged as a double-edged aspect of professional motivation. While empathy for animals sustained commitment to the profession, it simultaneously increased vulnerability to distress, particularly when suffering was perceived as preventable. As one participant reflected, “that love makes it harder.” Repeated exposure to animal pain and loss accumulated over time, contributing to emotional fatigue and gradual depletion of emotional resources.

In addition to external pressures, veterinary professionals described significant internalized stress related to uncertainty, responsibility, and self-doubt. Persistent concerns about treatment outcomes, end-of-life decisions, and professional adequacy extended beyond clinical encounters. One participant articulated this ongoing worry by asking, “What if the treatment plan does not work?” These internal processes sustained emotional engagement and compounded stress outside working hours.

Perceived gaps in caregivers’ knowledge and the influence of misinformation constituted another source of emotional labor. Participants described repeated efforts to correct unrealistic expectations regarding treatment outcomes, preventive care, and recovery timelines. One veterinary professional noted that some caregivers believed “we can do miracles,” which strained trust and complicated communication. The need to continuously justify clinical decisions intensified relational tension and emotional demands.

Finally, participants situated their experiences within broader societal attitudes toward animal welfare. Several veterinary professionals described a general lack of awareness regarding animal needs and rights, which heightened their sense of moral responsibility. One participant summarized this perception by stating, “There’s actually a general ignorance.” This broader context contributed to cumulative stress, as practitioners felt compelled not only to provide care but also to educate and advocate within an already emotionally demanding professional role.

#### 3.1.2 Ways to deal with stress

Veterinary professionals described coping not as the elimination of stress, but as ongoing efforts to manage and contain emotional strain arising from sustained job demands. Participants reported using a combination of interpersonal, behavioral, and emotion-focused strategies to remain functional within an emotionally demanding work environment.

Peer consultation and collegial support emerged as a primary coping resource. Participants described seeking advice from more experienced colleagues not only for clinical decision-making but also for guidance on communicating with caregivers during emotionally charged situations. One participant explained that they consulted colleagues about “how we should tell the pet owner.” Such informal debriefing processes helped reduce feelings of isolation and increased confidence when navigating morally or emotionally difficult encounters.

Boundary-setting was identified as a protective strategy aimed at limiting further emotional depletion. Participants emphasized the importance of establishing flexible but clear limits regarding availability, particularly in response to non-urgent after-hours requests. One veterinary professional noted the need to “set limits and tell them not to call for things that aren’t urgent.” These practices allowed practitioners to preserve personal time and reduce ongoing emotional exhaustion.

Physical self-care practices were described as essential for sustaining attention, emotional regulation, and clinical performance. Participants highlighted adequate sleep, regular meals, and short breaks as necessary for coping with long shifts and irregular schedules. As one participant stated, “sleep is always a necessity.” Such practices supported short-term recovery and helped prevent irritability and fatigue during demanding workdays.

Emotion-focused coping strategies were closely linked to meaning-making and professional identity. Several participants described reconnecting with animals outside of high-stakes clinical decision-making to restore emotional balance and reaffirm their motivation for the profession. One veterinary professional reflected that animals were part of their “rest and happiness.” Rather than eliminating stressors, these strategies enabled participants to sustain empathy and engagement despite ongoing emotional strain. Taken together, these coping strategies reflect efforts to manage and replenish emotional resources, allowing veterinary professionals to continue functioning within a persistently demanding occupational context.

#### 3.1.3 Needs for training

Veterinary professionals described training-related needs not in terms of technical competence, but as gaps in emotional, communicative, and ethical preparedness for daily practice. Participants consistently reported that their formal education emphasized biological and procedural knowledge, while offering limited preparation for managing emotionally complex interactions with caregivers and coping with the psychological demands of the profession.

Communication-related challenges were identified as a central area of unmet training needs. Participants noted that difficulties often arose not from clinical uncertainty, but from conveying unfavorable information, discussing financial limitations, or responding to caregivers’ emotional reactions. One veterinary professional remarked that “nobody teaches us how to deal with a crying pet owner.” Such experiences highlighted a perceived mismatch between academic training and the interpersonal realities of clinical work.

Emotional regulation and self-management skills were also described as insufficiently addressed during professional education. Participants reported difficulty processing emotionally intense experiences, particularly those involving euthanasia or preventable animal suffering. As one participant stated, “you leave an appointment after euthanasia and go right to the next one as if nothing happened.” The absence of structured guidance for navigating these transitions contributed to unresolved emotional strain and cumulative distress.

Participants further emphasized the lack of formal support mechanisms for reflecting on emotionally demanding cases. Several veterinary professionals described relying on trial-and-error learning to manage emotional burden, noting limited exposure to structured debriefing or supervised reflection during training. This absence was perceived as contributing to ongoing emotional exhaustion rather than facilitating adaptive coping.

Finally, participants identified limited training in animal behavior and caregiver psychology as an additional source of strain. Misinterpretation of animal stress signals and caregiver reactions was reported to exacerbate clinical tension and emotional difficulty. One participant observed that “a scared animal may be misinterpreted,” highlighting how gaps in behavioral understanding could intensify already challenging interactions. Taken together, these findings indicate that veterinary professionals perceive their training as insufficiently aligned with the emotional and relational demands of practice, contributing to sustained stress and reliance on informal, self-directed learning processes.

### 3.2 Companion animals’ caregivers

This theme focuses on companion animals’ caregivers as emotionally active participants in veterinary encounters whose reactions significantly shape clinical interactions. Veterinary professionals described a wide range of caregiver emotions, including apathy, anger, guilt, anxiety, and grief, reflecting the strength of the human–animal bond. Participants emphasized that responding to these emotional reactions constituted a substantial part of their daily work and contributed to the reciprocal emotional dynamics of veterinary care. The two subthemes; Responses and Support provided, exemplify how veterinary professionals perceive, manage, and respond to companion animals’ caregivers’ suffering, underscoring the emotional reciprocity intrinsic to veterinary care.

#### 3.2.1 Responses

Veterinary professionals interpreted caregivers’ reactions largely as grief-related responses shaped by attachment, loss, and perceived responsibility. Participants reported observing diverse emotional and behavioral patterns, ranging from avoidance and indifference to anger, remorse, and intense anxiety. While understanding these reactions was viewed as important for effective communication, managing them was also described as one of the most emotionally demanding aspects of practice.

Caregiver behaviors perceived as neglectful were frequently interpreted as avoidance-based coping rather than intentional disregard. Participants described situations in which delayed visits, missed follow-ups, or ignored recommendations reflected caregivers’ difficulty confronting the possibility of animal suffering or loss. One participant noted that caregivers sometimes “avoid coming to the clinic until it’s too late.” This avoidance complicated veterinary professionals’ emotional responses, creating tension between empathy and frustration.

Expressions of anger and hostility were commonly described in cases involving unfavorable outcomes or limited treatment options. Participants reported that such anger was often directed toward veterinary professionals, particularly when caregivers experienced fear, loss of control, or financial stress. As one veterinary professional stated, “the anger is directed at us.” These interactions heightened emotional demands and contributed to anticipatory stress during difficult consultations.

End-of-life decisions elicited particularly intense emotional reactions. Veterinary professionals described caregivers’ panic, guilt, and self-blame during discussions of euthanasia or terminal illness. One participant observed that caregivers repeatedly questioned whether they had done something wrong. These encounters positioned veterinary professionals at the intersection of clinical responsibility and shared emotional vulnerability, requiring continuous emotional regulation while maintaining professional functioning. Taken together, caregivers’ responses constituted a complex emotional landscape that veterinary professionals were required to navigate alongside clinical decision-making.

#### 3.2.2 Support provided

Veterinary professionals described providing emotional support to caregivers as an integral part of routine practice. Participants reported offering reassurance, empathy, and guidance alongside medical treatment, particularly during moments of acute distress. This support was framed as a practical response to caregivers’ emotional needs rather than as a separate or optional aspect of care.

Clear and transparent communication emerged as a primary strategy for supporting caregivers. Participants emphasized using simple language and visual explanations to reduce uncertainty and help caregivers understand diagnoses and treatment options. One veterinary professional explained that showing test results “makes it easier for them to accept the situation.” Such practices were perceived as facilitating trust and reducing anxiety during emotionally charged encounters.

Emotional regulation by veterinary professionals functioned as a stabilizing element in interactions with distressed caregivers. Participants reported consciously maintaining calm and composed behavior in response to anger, fear, or grief. One participant noted, “If I lose my calm, they lose theirs.” This approach helped de-escalate tension and maintain communication, though it required sustained emotional control.

Empathic presence and validation were also described as central components of support. Participants emphasized listening without judgment and acknowledging caregivers’ emotional experiences, particularly during moments of loss. One veterinary professional stated that simply expressing understanding “makes a difference.” Validation was perceived as helping caregivers feel recognized and supported during vulnerable moments.

In some cases, support extended beyond the immediate clinical encounter through brief follow-ups or additional guidance. While participants described these actions as meaningful for strengthening relationships with caregivers, they also acknowledged the emotional effort involved. As one participant reflected, “You give a piece of yourself every time.” These accounts illustrate how support provision, while central to veterinary practice, contributed to ongoing emotional demands.

## 4 Discussion

**Figure 3:**
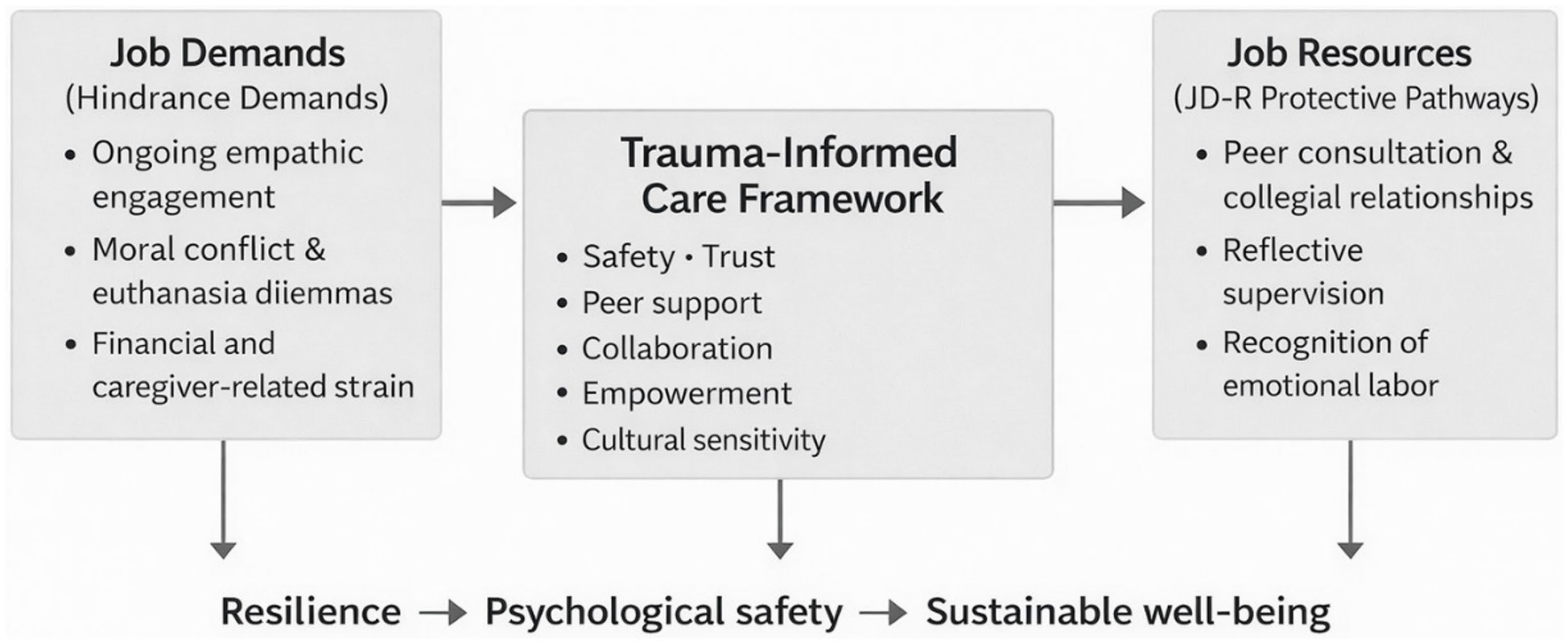
Trauma-informed organizational framework for veterinary occupational health illustrating how job demands and resource depletion lead to burnout and moral distress, and how peer support, reflective supervision, and the six principles of TIC (safety, trustworthiness, peer support, collaboration, empowerment, and cultural sensitivity) can foster resilience, psychological safety, and sustainable well-being.

This study examined veterinary professionals’ experiences of occupational stress, emotional strain, and coping and offers an exploratory, context-bound interpretation of these experiences through the framework of TIC. The findings suggest that veterinary practice is characterized by persistent moral and emotional demands, limited opportunities for recuperation, and uneven organizational support structures. Drawing on the Job Demands–Resources (JD–R) model (7) and Conservation of Resources (COR) theory (13), the findings indicate that sustained empathic engagement and moral conflict function as salient job demands that deplete emotional and cognitive resources, while limited social and institutional resources constrain recovery. Rather than making generalizable claims or positioning prior work as insufficient, this discussion proposes a conceptual framework for understanding how trauma-informed organizational practices may support resilience and psychological safety in veterinary contexts. These interconnections are summarized in Figure 3.

Although prior occupational health research has documented burnout and emotional exhaustion among veterinary professionals, much of this work has emphasized individual experiences and coping, while also acknowledging broader organizational and structural contributors to varying degrees. Consistent with qualitative and quantitative findings across caregiving professions, the present study suggests convergence with evidence from emergency medicine, palliative care, and child protection, where repeated exposure to death, grief, and ethical conflict is conceptualized as occupational trauma. Rather than redefining the field or challenging existing systemic perspectives, this study conceptualizes veterinary distress as sharing structural similarities with trauma-exposed caregiving work, extending JD–R and COR models toward a trauma-informed occupational perspective. In this sense, the contribution is theoretical and integrative rather than demonstrative.

### 4.1 Emotional and moral demands as persistent occupational stressors

Veterinary professionals’ accounts depict a work environment marked by continuous exposure to animal suffering, caregiver grief, and ethically constrained decision-making. These findings align with existing literature on compassion fatigue and moral distress in caregiving professions (11, 14). Within the JD–R framework, such experiences function as hindrance demands that consume energy without fostering growth. Participants’ descriptions of euthanasia, financial constraint, and caregiver conflict reflect moral incongruence between professional values and feasible action, a pattern previously linked to moral distress and burnout (8, 26).

What is theoretically noteworthy is not the presence of stressors per se, but their cumulative and relational nature. Veterinary professionals’ “dual caregiver” role intensifies emotional labor (9), as they manage both animal suffering and caregiver emotions. This finding converges with work in palliative and emergency care, where relational exposure is a key driver of occupational strain. COR theory helps explain how repeated moral and emotional demands initiate loss cycles that, without institutional replenishment, escalate into chronic strain.

### 4.2 Resource depletion, endurance, and organizational silence

Participants described a workplace culture that normalizes overextension and emotional self-reliance. Rather than interpreting this as individual resilience, the findings suggest the presence of a structural culture of endurance, comparable to patterns reported in other healthcare settings (15). From a COR perspective, the absence of formal recovery mechanisms perpetuates resource depletion, increasing vulnerability to burnout.

A trauma-informed interpretation offers a different reading: distress disclosure is constrained not by personal weakness but by insufficient psychological safety. TIC reframes recovery as an organizational responsibility, shifting attention from individual coping to structural interventions such as reflective supervision and debriefing. This does not replace JD–R or COR models but complements them, specifying how resources can be operationalized through trauma-sensitive practices (27).

### 4.3 Peer support as an informal but fragile resource

Peer consultation emerged as a critical relational resource, consistent with research highlighting social support as a buffer against emotional demands (28, 29). Participants’ narratives of shared meaning-making illustrate how collective sensemaking mitigates isolation and moral uncertainty. However, the informal nature of this support renders it uneven and dependent on personal initiative, limiting its protective capacity.

From a TIC perspective, peer support is a core principle that requires institutional scaffolding. This study suggests, rather than proves, that formalizing peer support mechanisms could transform an existing informal resource into a sustainable organizational practice, aligning relational resilience with occupational health objectives.

### 4.4 TIC as a complementary occupational health framework

TIC differs from traditional stress models by conceptualizing repeated exposure to death, grief, and moral conflict as trauma-related rather than merely stressful. Emerging veterinary literature has begun to acknowledge trauma-informed approaches, although applications remain limited and fragmented. The present study does not claim to introduce TIC to veterinary medicine but offers a contextualized application that integrates TIC principles with established OHP frameworks.

The six TIC principles, safety, trustworthiness, peer support, collaboration, empowerment, and cultural sensitivity (17), map closely onto constructs such as psychological safety and participatory climate (15). This convergence suggests theoretical compatibility rather than novelty, positioning TIC as a lens that deepens understanding of how organizational resources operate in trauma-exposed work.

### 4.5 Türkiye context and analytic transferability

The Türkiye context is characterized by rapid growth in companion-animal ownership, financial constraints, and limited institutional mental health supports. These contextual features likely intensify moral conflict and emotional labor, shaping how stress is experienced and managed. While some findings, such as financial constraint and informal coping, may be context-specific, others (euthanasia-related distress, caregiver grief, reliance on peer support) appear analytically transferable to other veterinary and caregiving contexts.

Accordingly, the findings should be interpreted as conceptually rather than statistically generalizable, offering insights into mechanisms that may manifest differently across cultural and institutional settings.

### 4.6 Limitations and future directions

This study is exploratory and based on a small, purposive sample, which limits statistical generalization. The male-dominated sample may underrepresent gendered dimensions of emotional labor, and participants who volunteered may have had heightened awareness of occupational stress, introducing selection bias. Accordingly, the findings should be interpreted as analytically transferable rather than context-specific, as the mechanisms identified, such as emotional labor, moral conflict, resource depletion, and relational trauma exposure, are theoretically relevant to veterinary practice beyond Türkiye and to other caregiving contexts, including large-animal and mixed clinical settings. However, policy-oriented implications derived from this study should be interpreted with caution, as governance structures, professional regulation, and occupational health responsibilities differ substantially across countries and veterinary sectors. Accordingly, trauma-informed policy recommendations are intended as adaptable principles rather than uniform prescriptions, requiring alignment with local regulatory frameworks and institutional capacities. Future research should examine trauma-informed interventions longitudinally and across diverse cultural and institutional contexts and include caregiver perspectives to further test and refine the transferability of these mechanisms.

## 5 Conclusion

Veterinary practice is a high-demand occupation that exists at the confluence of compassion, ethics, and trauma. This study illustrates that veterinary professionals’ discomfort is not only an individual inability to cope, but rather a foreseeable outcome of systematic exposure to moral and emotional pressures without sufficient resource replenishment. This study proposes a reconceptualization of TIC as an organizational-level occupational health paradigm, therefore expanding existing models of workplace stress to include the emotional dimensions of caring labor.

A trauma-informed workplace culture that fosters safety, trust, and cooperation may alleviate burnout, increase compassion satisfaction, and maintain professional engagement. Ultimately, caring for veterinary professionals as professionals who have been exposed to trauma on the job is not only a question of psychological care, but also of moral and organizational duty.

## Data Availability

The original contributions presented in the study are included in the article/supplementary material, further inquiries can be directed to the corresponding author.
